# Trends in the Utilization of Teleneurology and Other Healthcare Resources Prior to and During the COVID-19 Pandemic in an Urban, Tertiary Health System

**DOI:** 10.3389/fneur.2022.834708

**Published:** 2022-02-11

**Authors:** Benjamin R. Kummer, Parul Agarwal, Chloe Sweetnam, Jessica Robinson-Papp, Leah J. Blank, Ilana Katz Sand, Georges Naasan, Christina A. Palmese, Joohi Jimenez-Shahed, Jihan Grant, Shanna Patterson, Alison Navis, Laura K. Stein, Nathalie Jetté

**Affiliations:** ^1^Department of Neurology, Icahn School of Medicine at Mount Sinai, New York, NY, United States; ^2^Clinical Informatics, Mount Sinai Health System, New York, NY, United States; ^3^Department of Population Health Science and Policy, Icahn School of Medicine at Mount Sinai, New York, NY, United States

**Keywords:** underserved, telemedicine, teleneurology, telehealth, sociodemographic, equity, disparities, loss to follow-up

## Abstract

**Background:**

Patient groups traditionally affected by health disparities were less likely to use video teleneurology (TN) care during the initial COVID-19 pandemic surge in the United States. Whether this asymmetry persisted later in the pandemic or was accompanied with a loss of access to care remains unknown.

**Methods:**

We conducted a retrospective cohort study using patient data from a multicenter healthcare system in New York City. We identified all established pediatric or adult neurology patients with at least two prior outpatient visits between June 16th, 2019 and March 15th, 2020 using our electronic medical record. For this established pre-COVID cohort, we identified telephone, in-person, video TN or emergency department visits and hospital admissions for any cause between March 16th and December 15th, 2020 (“COVID period”). We determined clinical, sociodemographic, income, and visit characteristics. Our primary outcome was video TN utilization, and our main secondary outcome was loss to follow-up during the COVID period. We used multivariable logistic regression to model the relationship between patient-level characteristics and both outcomes.

**Results:**

We identified 23,714 unique visits during the COVID period, which corresponded to 14,170 established patients from our institutional Neurology clinics during the pre-COVID period. In our cohort, 4,944 (34.9%) utilized TN and 4,997 (35.3%) were entirely lost to follow-up during the COVID period. In the adjusted regression analysis, Black or African-American race [adjusted odds ratio (aOR) 0.60, 97.5%CI 0.52–0.70], non-English preferred language (aOR 0.49, 97.5%CI 0.39–0.61), Medicaid insurance (aOR 0.50, 97.5%CI 0.44–0.57), and Medicare insurance (aOR 0.73, 97.5%CI 0.65–0.83) had decreased odds of TN utilization. Older age (aOR 0.98, 97.5%CI 0.98–0.99), female sex (aOR 0.90 97.5%CI 0.83–0.99), and Medicaid insurance (aOR 0.78, 0.68–0.90) were associated with decreased odds of loss to follow-up.

**Conclusion:**

In the first 9 months of the COVID-19 pandemic, we found sociodemographic patterns in TN utilization that were similar to those found very early in the pandemic. However, these sociodemographic characteristics were not associated with loss to follow-up, suggesting that lack of TN utilization may not have coincided with loss of access to care.

## Introduction

In the first and second quarters of 2020, the global public health emergency caused by the novel coronavirus (coronavirus disease 2019 or COVID-19) promoted widespread adoption of telemedicine and teleneurology (TN) as patients and providers sought to minimize virus transmission and preserve access to neurological care worldwide (1–6). Building on the limited uptake of both telemedicine and TN preceding the COVID-19 pandemic, (7, 8) the COVID-19 crisis saw rapid increases in acceptance among neurologists for TN, which firmly established the latter as a viable care model in neurological populations (9). Despite the widespread adoption of TN and non-neurological telemedicine during the COVID-19 pandemic, several authors have noted sociodemographic differences in access to telemedicine during this period, raising the possibility of inequitable care (10–14) and potentiation of existing health disparities.

Neurological diseases are costly and often chronic conditions (15, 16). As such, the possibility of losing access to care for patients with neurological conditions constitutes a series of particularly impactful social, public health, and economic problems. Recent work has demonstrated that older, non-English speaking, Medicaid-insured, (17) and Black or African-American patients (14) are more likely to utilize telephone over video TN during the early COVID-19 pandemic in the US. However, few studies evaluate healthcare utilization beyond telephone or video TN visits, examine income and medical comorbidity as a sociodemographic characteristic, and analyze follow-up periods longer than the early stages of the pandemic (5, 14, 17). Additionally, no studies examine the degree to which patients lost access to their neurological providers during the public health emergency and may have turned to emergency or hospital care as stop-gap solutions, thereby not fully addressing larger-scale questions regarding the relationships between health resource utilization and global access to care for neurological patients during the COVID-19 pandemic.

We therefore sought to investigate the sociodemographic characteristics associated with video-only TN and multiple other measures of healthcare resource utilization over several months in a diverse patient cohort. Such measures of utilization included telephone visits, emergency department (ED) visits, hospital admissions, and loss to follow-up during the COVID-19 pandemic. This period spanned both the initial surge of the pandemic, in which in-person visits were infrequent, to later stages where in-person visits resumed. We hypothesized that (1) patients who did not have any TN visits were more likely to be older, non-White, non-English speaking, non-commercially insured, have greater medical comorbidity, live in areas with lower mean household incomes, and seek care through ED visits or hospital admissions for care during the COVID pandemic than patients that had a TN visit. We additionally hypothesized that (2) patients who were entirely lost to follow-up during the pandemic had similar clinical and sociodemographic profiles as patients that did not have a TN visit during the COVID pandemic.

## Materials and Methods

### Data Source and Cohort Identification

This was a retrospective study using patient data from the Mount Sinai Health System, an urban, academic, tertiary-care, six-hospital health system serving a diverse population in and around the New York City area. We used our institutional data warehouse (Epic ® Caboodle, Epic Systems Corp., Verona, WI USA), to identify all patients with a pediatric or adult neurology provider between June 16^th^, 2019 and March 15^th^, 2020. This time interval was defined as the “pre-COVID period,” with March 16^th^, 2020 marking the beginning of our Department's administrative procedures to mitigate virus transmission risk in the setting of the emerging public health threat related to the pandemic and the issued shelter-in-place order in New York State. These procedures consisted of converting scheduled in-person visits to video TN visits or deferral of care and have been described elsewhere (2).

Because patients with only one visit may have been seen for one-time consultations and did not necessarily represent established patients in our practices, we chose to include only patients that had two or more outpatient neurology visits during the pre-COVID period. We further excluded procedure-only visits, inter-professional electronic consultation visits, erroneous or no-show visits, and visits with non-physician or nurse-practitioner staff (e.g., social workers, pharmacists, or nutritionists). Using a field from our data warehouse that specified the provider's requested length of follow-up, we excluded patients who had a specified follow-up date during any pre-COVID visit that was outside the defined study period.

### Outcomes

Our primary outcome was TN utilization, defined as the presence of one or more video TN visits. We separated TN from telephone visits in order to better appreciate the sociodemographic differences that were already reported between users of both modalities (5, 14, 17). Our main secondary outcome was loss to follow-up, defined as the absence of any visits of any type during the COVID period. Other secondary outcomes included one or more telephone, office, ED or hospital admission visits during the COVID period. Use of all patient data for this study was approved by the Mount Sinai Institutional Review Board, who waived the requirement for informed consent.

For our study cohort, we identified all TN visits, telephone visits, and in-person office visits with a pediatric or adult neurology provider across our entire health system between March 16th and December 15^th^, 2020 (defined as the “COVID period”). TN was defined as comprising video visits only, based on the Centers for Medicare and Medicaid Services' definition of telehealth, which excludes audio-only telephone communications between providers and patients (18). We also identified all ED visits and hospital admissions for any cause to any Mount Sinai-affiliated hospitals in the COVID period. ED visits that subsequently became hospital admissions were counted as one instance of each visit type. Because technical problems, when they did arise, frequently led to TN visits being converted to a telephone visit, we excluded TN visits that occurred on the same date as a telephone visit with the same provider.

### Measurements

We determined demographic characteristics including age, sex, race, primary insurance coverage, ZIP code of primary residence, and preferred language, which was dichotomized to English or non-English. All socio-demographic characteristics were determined using the patient's first pre-COVID visit. Using 2018 income tax return data from the US Internal Revenue Service, we calculated household annual gross income (AGI) for each patient's ZIP code by dividing the total ZIP code AGI figure by the number of tax returns. We used the first 10 medical diagnoses listed in the patient's medical history at their first pre-COVID period visit to derive a Charlson-Deyo comorbidity index for each patient (19). We also determined visit related information, including date, type, provider, and visit diagnosis. To facilitate analysis of patient-level characteristics, only pre-COVID period visit diagnoses were incorporated into the analysis. Two authors with experience in administrative datasets (NJ, BRK) categorized all visit diagnosis codes into clinically meaningful groups (Supplementary Table 1).

### Statistical Analysis

Standard descriptive statistics (mean, median, interquartile range, percentages) were conducted based on type of variable. We compared continuous variables using the Student's *t*-test or Wilcoxon rank-sum tests, and categorical variables using the chi-squared as appropriate. Multiple imputation of unknown race data was conducted using the fully conditional method (20). Ten repetitions were performed to generate 10 imputed datasets.

We used multivariable logistic regression to model the relationships between clinical and sociodemographic characteristics and both TN utilization and loss to follow-up. The multivariable regression analysis was adjusted for all clinical and sociodemographic characteristics, which were entered into both models using the all-at-once approach. Multivariable regressions were performed on 10 imputed datasets and corresponding odds ratios and confidence intervals were appropriately combined. The cutoff for significance was set to 0.025, after using Bonferroni correction to adjust both regression analyses for multiple comparisons (21). All statistical analyses were performed using SAS software (SAS Institute Inc., Cary, NC, USA).

### Sensitivity Analysis

To account for the effect of loss to follow-up, which may have skewed the composition of the non-TN utilizing patient population and thereby exaggerated or understated differences between non-TN utilizing and utilizing populations, we conducted a sensitivity analysis in which we excluded patients who were lost to follow-up and then compared TN and non-TN utilizing patient groups.

## Results

**Figure 1:**
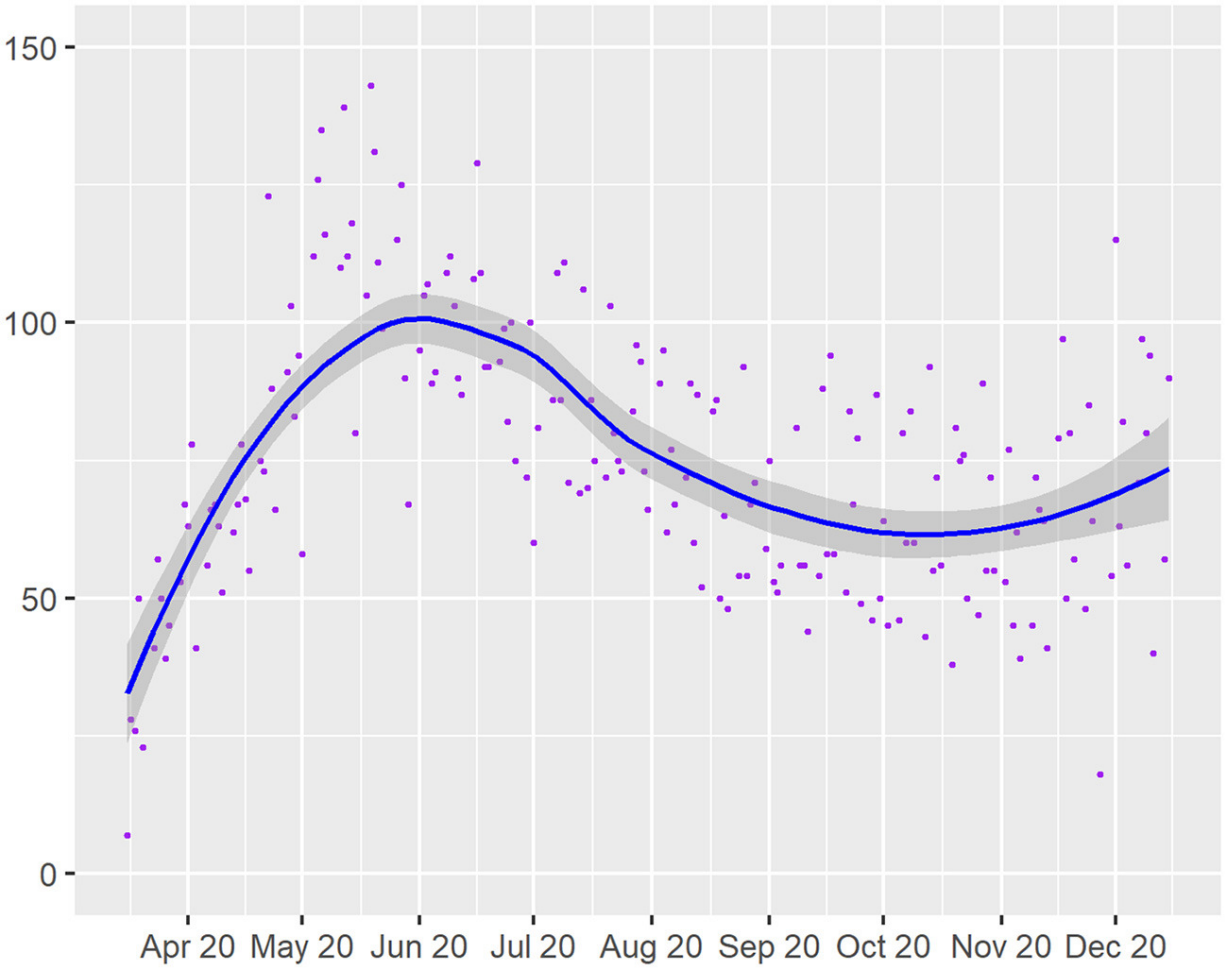
Daily teleneurology visit counts (purple dots) over time during the 9-month COVID study period. Curve of best fit is shown in blue, and 95% confidence estimate intervals for best fit curve are shown in shaded areas.

We identified 43,854 and 23,714 visits in the 9-month pre-COVID and COVID periods, respectively. The pre-COVID period visits corresponded to 14,170 established patients from our institutional neurology clinics. Of these established patients, the median age was 59 years (IQR 39-72), 60.4% were female, 41.8% were commercially insured, and the median household AGI was $86,910 (IQR $ 53,908–$179,965). During the COVID period, 34.9% had one or more TN visits and 35.3% were lost to follow-up (Table 1). The temporal distribution of daily TN visits is displayed in Figure 1.

**Table 1:** Patient characteristics, stratified by teleneurology utilization during the COVID period.

**Characteristic**	**Overall**	**Had a TN visit**	**Did not have a TN visit**	***P*-value**
	**(*N* = 14,170)**	**(*N* = 4,944 (34.9%))**	**(*N* = 9,226 (65.1%))**	
Age, median (IQR), years	59 (39–72)	54 (37–69)	61 (41–74)	<0.0001
Female sex	8,562 (60.4)	3,058 (61.8)	5,504 (59.7)	0.0109
**Race** ^*^				<0.0001
Native American	25 (0.18)	10 (0.2)	15 (0.2)	
Asian	564 (4.0)	197 (4.0)	367 (4.0)	
Black or African American	2,245 (15.8)	617 (12.5)	1,628 (17.6)	
Pacific Islander	70 (0.5)	17 (0.4)	53 (0.6)	
Not reported	3,374 (23.8)	879 (17.8)	2,495 (27.0)	
White	7,894 (55.7)	3,225 (65.2)	4,669 (50.6)	
**Preferred language**				<0.0001
Non-English	1,006 (7.1)	146 (2.9)	860 (9.3)	
English	13,080 (92.3)	4,785 (96.7)	8,295 (89.9)	
Unknown	84 (0.6)	13 (0.3)	71 (0.8)	
**Insurance coverage**				<0.0001
Commercial	5,919 (41.8)	2,603 (52.6)	3,316 (35.9)	
Medicaid	1,859 (13.1)	392 (7.9)	1,467 (15.9)	
Medicare	5,447 (38.4)	1,608 (32.5)	3,839 (41.6)	
Other	351 (2.5)	129 (2.6)	222 (2.4)	
Unknown	594 (4.2)	212 (4.3)	382 (4.1)	
ZIP code AGI per household, median (IQR), $US	86,910 (53,908–179,965)	98,802 (60,604–220,597)	79,321 (52,981–173,596)	<0.0001
**Charlson-Deyo comorbidity index**				<0.0001
0	9,121 (64.4)	3,544 (71.7)	5,577 (60.4)	
1	2,834 (20.0)	842 (17.0)	1,992 (21.6)	
2	1,091 (7.7)	298 (6.0)	793 (8.6)	
≥3	1,124 (7.9)	260 (5.3)	864 (9.4)	
Health utilization during COVID19 period**				
≥1 office visit	4,577 (32.3)	1,645 (33.3)	2,932 (31.8)	0.0701
≥1 telephone visit	2,093 (14.8)	707 (14.3)	1,386 (15.0)	0.2479
≥1 ED visit	1,314 (9.3)	351 (7.1)	963 (10.4)	<0.0001
≥1 hospital admission	885 (6.3)	304 (6.1)	581 (6.3)	0.7276
Lost to follow-up	4,997 (35.3)	0 (0.0)	4,997 (54.2)	<0.0001

*Data are reported as N (%) unless otherwise noted*.

* Race variable is imputed as described in Methods. **Percentages may not add to 100% since categories are not mutually exclusive.

*COVID19, coronavirus disease 2019; IQR, interquartile range; AGI, adjusted gross income; ED, emergency department*.

### Teleneurology Utilization

Patients who utilized TN during the COVID period were significantly younger (median 54.0 vs. 61.0 years, *p* < 0.0001), and more likely to be female (61.8 vs. 59.7%, *p* = 0.01087) and White (65.2 vs. 50.6%, *p* < 0.0001) than their non-TN utilizing counterparts. Compared to the latter group, patients who utilized TN were also more likely to have commercial insurance (52.6 vs. 35.9%, *p* < 0.0001), reside in a ZIP code with higher household AGI (median $98,802 vs. 79,321, *p* < 0.0001), and list English as their preferred language (96.7 vs. 89.9%, *p* < 0.0001). Finally, patients who utilized TN were also less likely to have one or more ED visits during the COVID period than patients who did not have any TN visits (7.1 vs. 10.4%, *p* < 0.0001) (Table 1). Stratified pre-COVID visit diagnoses are shown in Supplementary Table 2.

The results of the sensitivity analysis were similar and more accentuated than in the primary analysis. The two exceptions to this trend were that the proportion of female patients was no longer significantly different between TN utilizing and non-TN utilizing patients (61.8 vs. 61.4%, *p* = 0.65). In addition, patients that utilized TN during the COVID period were significantly less likely to have one or more telephone, office, ED, or hospital admission visits than patients who did not utilize TN (Table 2).

**Table 2:** Sensitivity analysis of TN utilization during the COVID period, excluding patients lost to follow-up.

**Characteristic**	**Had a TN visit (*N* = 4,944)**	**Did not have a TN visit (*N* = 4,229)**	***P*-value**
Age, median (IQR), years	54 (37–69)	65 (52–76)	<0.0001
Female sex	3,058 (61.8)	2,596 (61.4)	0.6465
**Race** ^*^			<0.0001
Native American	10 (0.2)	9 (0.2)	
Asian	197 (4.0)	156 (3.7)	
Black or African American	617 (12.5)	805 (19.0)	
Pacific Islander	17 (0.4)	32 (0.8)	
Not reported	879 (17.8)	1,219 (28.8)	
White	3,225 (65.2)	2,008 (47.5)	
**Preferred language**			<0.0001
Non-English	146 (2.9)	478 (11.3)	
English	4,785 (96.7)	3,730 (88.2)	
Unknown	13 (0.3)	21 (0.5)	
**Insurance coverage**			<0.0001
Commercial	2,603 (52.6)	1,053 (24.9)	
Medicaid	392 (7.9)	779 (18.4)	
Medicare	1,608 (32.5)	2,153 (50.9)	
Other	129 (2.6)	70 (1.6)	
Unknown	341 (6.9)	244 (5.7)	
ZIP code AGI per household, median, $US	98,802 (60,604–220,597)	74,949 (50,953–173,596)	<0.0001
**Charlson-Deyo comorbidity index**			<0.0001
0	3,544 (71.7)	2,271 (53.7)	
1	842 (17.0)	1,025 (24.2)	
2	298 (6.0)	453 (10.7)	
≥3	260 (5.3)	480 (11.3)	
Health utilization during COVID19 period^**^			
≥1 office visit	1,645 (33.3)	2,932 (69.3)	<0.0001
≥1 telephone visit	707 (14.3)	1,386 (32.8)	<0.0001
≥1 ED visit	351 (7.1)	963 (22.8)	<0.0001
≥1 hospital admission	304 (6.1)	581 (13.7)	<0.0001

*Data are reported as N (%), unless otherwise noted*.

* *Race variable is imputed as described in Methods*.

** *Percentages may not add to 100% since categories are not mutually exclusive*.

*TN, teleneurology; COVID19, coronavirus disease 2019; IQR, interquartile range; ED, emergency department; AGI, annual gross income*.

In the multivariable regression analysis, Black or African-American race (aOR 0.60, 97.5%CI 0.52–0.70), non-English preferred language (aOR 0.49, 97.5%CI 0.39–0.61), and Medicaid (aOR 0.50, 97.5%CI 0.43–0.58) or Medicare (aOR 0.73, 97.5%CI 0.65–0.83) insurance were significantly associated with lower odds of utilizing TN. Several individual pre-COVID period visit diagnoses (e.g., dementia, epilepsy, and demyelinating disorders) were associated with higher odds of having a subsequent TN visit during the COVID period. Other diagnoses (e.g., musculoskeletal and circulatory disorders) were associated with lower odds (Table 3).

**Table 3:** Multivariable analysis of characteristics associated with TN utilization during the COVID period.

**Characteristic**	**Adjusted OR**	**97.5% CI**	***P*-value**
Age	1.00	0.99–1.00	0.185
Female sex	1.03	0.94–1.13	>0.99
**Race**			
Native American	0.78	0.27–2.26	>0.99
Asian	1.02	0.81–1.29	>0.99
Black or African-American	0.60	0.52–0.70	<.0001
Pacific Islander	0.64	0.31–1.29	0.3046
Not reported	0.65	0.57–0.75	<.0001
**Preferred language**			
Non-English	0.49	0.39–0.61	<.0001
Unknown	0.32	0.16–0.65	0.0006
ZIP code AGI per household	1.00	1.00–1.00	0.6472
**Insurance coverage**			
Medicaid	0.50	0.43–0.58	<.0001
Medicare	0.73	0.65–0.83	<.0001
Unknown	0.89	0.71–1.10	0.4410
Other	1.09	0.83–1.43	0.9378
Charlson-Deyo comorbidity index	1.03	0.96–1.02	0.76
**Pre-COVID period visit diagnosis**			
Neoplasms	0.83	0.83–1.60	0.6476
Endocrine disorders	1.00	0.74–1.35	>0.99
Dementia and delirium	1.64	1.30–2.07	<.0001
Mental (excluding neurodevelopmental) disorders	1.83	1.49–2.24	<.0001
Neurodevelopmental, behavioral and emotional disorders with childhood/adolescent onset	1.59	1.22–2.06	<.0001
Systemic atrophies of the CNS	2.01	1.27–3.17	0.0014
Extrapyramidal and movement disorders	2.44	2.11–2.81	<.0001
Demyelinating CNS disorders	6.41	5.46–7.52	<.0001
Epilepsy	1.52	1.31–1.78	<.0001
Migraine and other headache disorders	2.16	1.88–2.48	<.0001
Stroke and cerebrovascular disorders	0.90	0.73–1.09	0.4218
Sleep and other neurological disorders	0.98	0.79–1.21	> 0.99
Neuromuscular disorders	0.88	0.75–1.04	0.188
Eye, ear, and adnexal disorders	0.63	0.47–0.85	0.001
Circulatory (excluding cerebrovascular) disorders	0.67	0.46–0.98	0.035
Infectious disorders	1.24	0.85–1.80	0.412
Respiratory, digestive, and skin disorders	0.86	0.67–1.11	0.3812
Musculoskeletal and connective tissue disorders	0.98	0.86–1.13	>0.99
Abnormal clinical and laboratory findings	0.99	0.86–1.14	>0.99
Symptoms and signs of nervous and musculoskeletal systems	0.73	0.59–0.90	0.0016
Symptoms and signs of behavioral, cognitive systems	0.75	0.63–0.91	0.001

*Regression model references are as follows: White (race); English (preferred language); commercial insurance (insurance coverage). Model uses imputed race variables*.

*TN, teleneurology; OR, odds ratio; CI, confidence interval; AGI, adjusted gross income; CNS, central nervous system*.

### Secondary Outcomes

Patients who were lost to all follow-up during the COVID period were significantly more likely to be younger (median 56 vs. 60 years, *p* < 0.0001), male (41.8 vs. 38.4%, *p* < 0.0001), and list a preferred language other than English (7.6 vs. 6.8%, *p* < 0.0001) than patients that were not lost to follow-up. Compared to the latter group, patients who were lost to follow-up were also more likely to be commercially-insured (44.9 vs. 39.9%, *p* < 0.0001), live in lower-income ZIP codes (median AGI $84,311 vs. 88,124, *p* = 0.0253), and have a Charlson-Deyo comorbidity index of 1 or lower (85.5 vs. 83.7%, *p* = 0.0028) (Table 4). Stratified pre-COVID visit diagnoses are shown in Supplementary Table 3.

**Table 4:** Patient characteristics, stratified by loss to follow-up during the COVID period.

**Characteristic**	**Lost to follow-up (*N* = 4,997)**	**Not lost to follow-up (*N* = 9,173)**	***P*-value**
Age, median (IQR), years	56 (31–72)	60 (43–72)	<0.0001
Female sex	2,908 (58.2)	5,654 (61.6)	<0.0001
**Race** ^*^			<0.0001
Native American	6 (0.1)	19 (0.2)	
Asian	211 (4.2)	353 (3.8)	
Black or African American	823 (16.5)	1,421 (15.5)	
Pacific Islander	21 (0.4)	50 (0.5)	
Not reported	1,276 (25.5)	2,098 (22.9)	
White	2,661 (53.3)	5,233 (57.1)	
**Preferred language**			<0.0001
English	4,565 (91.3)	8,515 (92.8)	
Non-English	382 (7.6)	624 (6.8)	
Unknown	50 (1.0)	34 (0.4)	
**Insurance coverage**			<0.0001
Commercial	2,246 (44.9)	3,660 (39.9)	
Medicaid	685 (13.7)	1,170 (12.7)	
Medicare	1,691 (33.8)	3,742 (40.8)	
Other	155 (3.1)	201 (2.2)	
Unknown	220 (4.4)	400 (4.4)	
ZIP code AGI per household, median, $US	84,311 (53,526–179,965)	88,124 (56,093–182,162)	0.0253
**Charlson-Deyo comorbidity index**			0.0028
0	3,306 (66.2)	5,815 (63.4)	
1	967 (19.3)	1,867 (20.3)	
2	340 (6.8)	751 (8.2)	
≥3	384 (7.7)	740 (8.1)	

*Data are reported as N (%), unless otherwise noted*.

* *Race variable is imputed as described in Methods*.

*COVID19, coronavirus disease 2019; IQR, interquartile range; AGI, annual gross income*.

In the multivariable regression analysis, the only characteristics that were significantly associated with a greater odds of loss to follow-up were unknown preferred language (aOR 3.08, 97.5%CI 1.82–5.19) and a pre-COVID diagnosis of eye and ear disorders (aOR 1.37, 97.5%CI 1.08–1.74) (Table 5). By contrast, older age (aOR 0.98, 97.5%CI 0.98–0.99), female sex (aOR 0.90, 97.5%CI 0.83–0.99), and Medicaid (aOR 0.78, 97.5%CI 0.68–0.90) insurance were associated with significantly lower odds of loss to follow-up. Similar to the primary regression analysis, several individual pre-COVID visit diagnoses (e.g., dementia and epilepsy) were also associated with lower odds of loss to follow-up (Table 5).

**Table 5:** Multivariable analysis of patient characteristics associated with loss to follow-up during the COVID period.

**Characteristic**	**Adjusted OR**	**97.5% CI**	***P*-value**
Age	0.98	0.98–0.99	<.0001
Female sex	0.90	0.83–0.99	0.02
**Race**			
Native American	0.58	0.15–2.31	0.74
Asian	0.94	0.74–1.20	>0.99
Black or African American	1.11	0.97–1.27	0.16
Pacific Islander	0.65	0.33–1.30	0.33
Not reported	1.12	0.98–1.27	0.11
**Preferred language**			
Non-English	1.15	0.96–1.37	0.15
Unknown	3.08	1.82–5.19	<.0001
ZIP code AGI per household	1.00	1.00–1.00	>0.99
**Insurance coverage**			
Medicaid	0.78	0.68–0.90	<.0001
Medicare	0.91	0.81–1.03	0.20
Unknown	0.97	0.78–1.20	0.99
Other	1.12	0.86–1.45	0.69
Charlson-Deyo comorbidity index	0.98	0.95–1.01	0.20
**Pre-COVID period visit diagnosis**			
Neoplasms	0.77	0.57–1.05	0.11
Endocrine, nutritional and metabolic diseases	0.90	0.69–1.18	0.76
Dementia and delirium	0.81	0.65–1.01	0.07
Mental (excluding neurodevelopmental) disorders	0.68	0.55–0.85	<.0001
Neurodevelopmental, behavioral and emotional disorders with childhood/adolescent onset	0.57	0.44–0.73	<.0001
Systemic atrophies of the CNS	0.71	0.46–1.10	0.16
Movement disorders	0.34	0.29–0.40	<.0001
Demyelinating CNS disorders	0.18	0.15–0.21	<.0001
Epilepsy	0.51	0.44–0.59	<.0001
Migraine and other headache disorders	0.42	0.37–0.48	<.0001
Stroke and cerebrovascular disorders	1.07	0.90–1.26	0.75
Sleep disorders	0.99	0.82–1.19	>0.99
Neuromuscular disorders	1.02	0.88–1.18	>0.99
Eye, ear, and adnexal disorders	1.37	1.08–1.74	0.01
Circulatory disorders	0.98	0.72–1.32	>0.99
Infectious disorders	0.77	0.54–1.09	0.18
Respiratory, digestive, and skin disorders	0.64	0.50–0.81	<.0001
Musculoskeletal disorders	0.76	0.67–0.87	<.0001
Abnormal clinical or laboratory findings, not elsewhere classified	0.76	0.65–0.90	0.55
Symptoms and signs of nervous and musculoskeletal systems	0.94	0.82–1.07	0.01
Symptoms and signs of cognition, perception, emotional state and behavior	1.25	1.04–1.49	<.0001

*Regression model references are as follows: White (race); English (preferred language); commercial insurance (insurance coverage)*.

*Model uses imputed race variables*.

*TN, teleneurology; OR, odds ratio; CI, confidence interval; AGI, adjusted gross income; CNS, central nervous system*.

Patients who had one or more telephone visits during the COVID period were significantly more likely to be older, of Black or African-American race, and have a non-English preferred language than patients who did not have any telephone visits. Compared to the latter group, telephone visit-utilizing patients were also more likely to be Medicare- or Medicaid-insured, have a higher degree of medical comorbidity, and have one or more office, ED, or hospital visits during the pandemic (Supplementary Table 4). We found similar but more attenuated differences in office visit utilization than those observed for telephone visits (Supplementary Table 5). In contrast, we found similar but more pronounced differences in use of ED visit and hospitalizations during the COVID period, with two exceptions. Notably, patients who had presented to the ED visit were significantly more likely to be female and not use TN than patients who did not have an ED visit. Additionally, patients who were hospitalized were significantly more likely to be male than patients who were not hospitalized (Supplementary Tables 6, 7).

## Discussion

In this retrospective study of over 14,000 established neurology clinic patients from a large, urban, multicenter, tertiary care health system in the 9 months prior to and following the onset of the COVID-19 pandemic in New York City, we found differences in TN utilization according to age, race, income, insurance coverage, comorbidity, preferred language, and utilization of ED care. We found that only non-English preferred language, Black or African-American race, and Medicare or Medicaid insurance coverage were significantly associated with decreased odds of TN utilization during the pandemic. Furthermore, we found that loss to follow-up differed according to age, sex, income, preferred language, and medical comorbidity. Interestingly, older age, female sex, and Medicaid insurance were significantly associated with a decreased odds of loss to follow-up. Additionally, we found that patients that had telephone, office, ED, or hospitalization visits during the COVID-19 pandemic were more likely to be Medicare-insured and harbor greater medical comorbidity than patients who did not use these care modalities.

The 34.9% rate of TN utilization we found is similar to that found in two recent studies, (14, 17) although the 14.8% telephone utilization rate we report is significantly lower than one study. This finding may be related to our design of excluding patients with only one visit, as well as these preceding studies' smaller cohorts and earlier studied period during the COVID-19 pandemic. Our study builds on this prior work by including a longer follow-up period that includes both initial and later stages of the COVID-19 pandemic where in-person visits began occurring more regularly, and investigates multiple health utilization outcomes, including loss to follow-up. Furthermore, our study attempts to establish patterns of association between patient-level sociodemographic and clinical characteristics with TN utilization and loss to follow-up.

We had initially hypothesized that patients who did not have any TN visits would more likely be older, non-White, non-English speaking, non-commercially insured, have greater medical comorbidity, live in areas with lower household incomes, and seek care through ED visits or hospital admissions for care during the COVID pandemic than patients that had a TN visit.

Although non-TN utilizing population demonstrated all of the characteristics we had hypothesized, hospitalization rates were not different between TN and non-TN utilizing groups. We therefore could not accept our first hypothesis.

Despite this, many of our findings are consistent with prior investigations. Notably, studies of neurological patient populations during the early COVID-19 surge have demonstrated that Black or African-American, (5, 14) lower-income, (5) and Medicare- or Medicaid-insured (14) patients were less likely to complete TN video visits rather than telephone visits. In another comparable study, patients who had telephone visits instead of TN visits were more likely to be older, non-commercially insured than patients evaluated by TN, with a pediatric subgroup being more likely to be non-English speaking (17). Furthermore, two of the aforementioned studies were conducted in urban tertiary-care settings similar to ours, (5, 17) lending further credence to the generalizability of our results.

Similar studies in non-neurological populations (10, 22–28) have shown consistent results with ours, with one study from a large urban health system demonstrating that socially vulnerable populations were more likely to use ED care and office visits in favor of telemedicine care (10). It is interesting to note that multiple studies conducted prior to the COVID-19 pandemic have found that minority status was associated with increased odds of telemedicine utilization in comparison to White patient groups, (29–31) suggesting that our findings may be in part related to the extraordinary nature of the COVID-19 public health emergency. Nonetheless, taken together with results from previous studies, our findings underscore the presence of important asymmetries in TN access for traditionally disadvantaged patient populations during the COVID pandemic. These care asymmetries carry meaningful social consequences and require attention at a systemic level.

Importantly, our findings do not fully explain or identify the causes of the TN utilization asymmetries we observed. Contributing factors likely include existing, inter-related digital and socioeconomic inequalities in the US healthcare system that clearly preceded the COVID-19 crisis. This digital divide has been shown to disproportionately affect the most disadvantaged patients in society, including ethnic minority, (32) elderly, (33, 34) economically disadvantaged, (34, 35) non-English speaking, and low health literacy patient groups (36). Concerningly, technology access gaps persisted during the early and middle phases of the COVID-19 crisis, with patients from disadvantaged populations continuing to demonstrate poor utilization of both telehealth (37, 38) and digital technologies (39–41). Echoing the concerns with respect to global care equity raised by several authors during this period, (10, 12, 22, 34, 37, 40–43) our results, when taken together with the technological requirements of TN, may suggest that vulnerable patient populations may have been at a disadvantage during the larger shift to digital care platforms TN that occurred in the early COVID-19 pandemic.

However, the digital divide may not be the sole explanation for our results, particularly among Medicaid beneficiaries. At our institution, a diverse population of Medicaid-insured patients are treated in hospital-administered clinics by resident and fellow trainees under the supervision of attending neurologists (2). Because many trainees were deployed to inpatient services for COVID-related care during the first several months of the COVID surge (2) and only returned to in-person office visits in June 2020, Medicaid-insured patients were likely unable to find available providers during the initial 3 months of the COVID pandemic between March and May 2020. This return to office visit care, combined with a preference for office over TN care, may also partially explain why Medicaid-insured patients were significantly less likely to be lost to follow-up during the COVID period. Additionally, hospital-administered and faculty practice clinics may have differed in the degree of TN platform on-boarding and technical support that was provided to patients and their caregivers to encourage familiarity with TN care. This may have driven some of the decreased TN utilization among Medicaid-insured patients.

Additionally, the lack of integrated translator services in our institutional TN platform during the early COVID-19 pandemic period could have been the cause of low utilization among non-English speaking populations. While translator services were available during this period, they were not integrated into the official institutional TN platform and required providers to access the services *via* a separate but concurrent telephone communication. Providers' variable technology preferences and beliefs about telemedicine care may also have significantly influenced the degree of TN utilization.

Similar to patients who did not utilize TN during the 9-month COVID study period, we found that patients who were lost to follow-up were more likely to have a preferred language other than English and Medicaid insurance. Well-documented associations between limited health care access and reduced English proficiency, (42–46) Medicare or Medicaid insurance, and low income (47) may explain some of these commonalities. However, our second regression analysis suggests that neither language preference nor income were independently associated with loss of global access to care, and that patients with Medicaid insurance were in fact less likely than commercially-insured patients to be lost to follow-up during the pandemic (Table 5).

Despite some overlap between these two patient groups, we found significant sociodemographic differences. In comparison to patients who were not lost to follow-up, those who had no visits during the COVID period were more likely to be younger, commercially-insured, and have lower degrees of medical comorbidity. One explanation for this is that the latter population may have reflected the demographic makeup of patients who migrated out of the New York City area during the COVID-19 pandemic. Although little has been documented about this population's insurance coverage or degree of medical comorbidity, populations that migrated out of New York City have been shown to be relatively younger than populations that did not migrate (48). Additionally, patients who had few medical comorbidities may have been more likely to temporarily suspend their care than patients with greater comorbidities. Finally, this population may have also comprised patients who had less restrictive insurance plans or greater financial means and were therefore able to seek care at healthcare institutions other than ours in the New York City area during the COVID-19 pandemic.

The question of a potential relationship between TN utilization and loss to follow-up is also important for contextualizing our study's results with respect to both individual outcomes. While we could not establish that low TN utilization definitively caused insufficient or absent follow-up, we did find that nearly half of the patients who did not have a TN visit were also lost to follow-up during the COVID period, and vice-versa. Because we defined follow-up to include ED and hospital visits for both neurological and non-neurological reasons, it is unclear whether low TN utilization truly co-occurred with loss of outpatient neurological follow-up. Despite our finding that publicly-insured, Black or African-American, and non-English speaking patients were significantly less likely to utilize TN than their commercially-insured, White, and English-speaking counterparts, these same patient factors were not significantly associated with loss to follow-up, suggesting that such patients received care through non-TN modalities.

The likely explanation for this is our finding that non-TN utilizing patients were significantly more likely than TN-utilizing patients to seek ED care during the COVID period. Additionally, patients who had more than one ED visit during the COVID period were also more likely to belong to vulnerable populations than their counterparts who did not present to the ED. Taken together, these findings are consistent with existing studies demonstrating that patients that preferentially used EDs for care over telehealth during the early COVID surge were more likely to belong to minority populations (10, 24). Reassuringly consistent with a recent study, (27) these groups were also well-represented among patients that had one or more office visits during the pandemic period, suggesting that despite lower TN utilization, populations that are historically affected by health disparities may have been able to preserve their access to their neurological providers through in-person, office encounters (Supplementary Table 3).

### Limitations

This study was limited by several notable factors. First, the generalizability of our results may be limited, given the exceptional nature of the COVID-19 public health emergency and the resulting, unusually profound impacts on neurological care delivery. Our analysis also lacked granular sociodemographic characteristics such as providers' attitudes toward TN, patient domiciled status, access to caregivers or home assistance, and reliable access to Wi-Fi, smartphones, or computers. We also could not differentiate those patients that presented to the ED or were hospitalized for neurological complaints, or those who were completely lost to neurological follow-up but may have presented to the ED or been hospitalized for non-neurological conditions. Because we could not collect information relating to ED visits or hospitalizations at institutions other than ours from our clinical data warehouse, the rates of both these outcomes may have been understated. Additionally, our analysis did not incorporate text data, including follow-up plans from visit progress notes. We therefore could not use this information to identify patients who were directed to follow-up after the end of the study period. However, to partially address this limitation, we identified a likely subgroup of such patients by using a discrete but less reliably populated field in our data warehouse.

In this retrospective cohort analysis of TN utilization at an urban tertiary-care Medical Center before and during the COVID-19 pandemic, we found that TN utilization varied according to race, income, insurance, and preferred language. By contrast, differences in loss to follow-up varied according to different, and times opposite patterns in the same factors. Importantly, none of these sociodemographic factors, with the exception of Medicaid insurance coverage, were significantly associated with loss to follow-up. This may suggest that low TN utilization may have coincided with, but not necessarily translated to loss of follow-up during the pandemic. Finally, we also found that populations with low TN utilization were more likely to use ED visits for care, and both groups had significant sociodemographic overlap, raising the possibility that the two may be causally related.

Further studies should incorporate granular data such as measures of patient education, provider attitudes, and technological literacy into analyses of TN utilization in order to better understand the causes of our findings. Future TN investigations should also study the effects of TN utilization on neurological care outcomes, as well as the optimization of TN care access among patients from different sociodemographic groups.

## Data Availability Statement

The datasets presented in this article are not readily available because this would jeopardize patient privacy. De-identified and/or anonymized data may be made available upon request by qualified investigators with sponsorship by an institution. Determination and/or approval from the Institutional Review Board of the Icahn School of Medicine at Mount Sinai as well as the receiving institution will be required. Requests to access the datasets should be directed to Benjamin R. Kummer, benjamin.kummer@mountsinai.org.

## Ethics Statement

The studies involving human participants were reviewed and approved by Institutional Review Board of the Icahn School of Medicine at Mount Sinai. Written informed consent from the participants' legal guardian/next of kin was not required to participate in this study in accordance with the national legislation and the institutional requirements.

## Author Contributions

BK: conceptualized the study, obtained study data, analyzed study data, interpreted study data, drafted the manuscript, and revised the manuscript for key intellectual content. PA: obtained study data, analyzed study data, interpreted study data, and revised the manuscript for key intellectual content. CS, JR-P, LB, IK, GN, CP, JJ-S, JG, SP, AN, and LS: interpreted study data and revised the manuscript for key intellectual content. NJ: conceptualized study, interpreted study data, and revised the manuscript for key intellectual content. All authors contributed to the article and approved the submitted version.

## Conflict of Interest

BK has received consulting fees from MD Aware and serves on the scientific advisory board of Syntrillo. JJ-S has received research support from the Michael J Fox Foundation and Impax Laboratories; she has served as consultant for Medtronic, Signant Health, St. Jude Medical, Abbvie, Teva, Spark Therapeutics, and Revance; she has served on the data safety monitoring committee of Blue Rock Therapeutics. The remaining authors declare that the research was conducted in the absence of any commercial or financial relationships that could be construed as a potential conflict of interest.

## Publisher's Note

All claims expressed in this article are solely those of the authors and do not necessarily represent those of their affiliated organizations, or those of the publisher, the editors and the reviewers. Any product that may be evaluated in this article, or claim that may be made by its manufacturer, is not guaranteed or endorsed by the publisher.
