# Supplementary material for: Trends in the Utilization of Teleneurology and Other Healthcare Resources Prior to and During the COVID-19 Pandemic in an Urban, Tertiary Health System
Source: Front Neurol. 2022 Feb 11;13:834708. doi: 10.3389/fneur.2022.834708 (PMC8873082; doi:10.3389/fneur.2022.834708)
Supplement: Supplementary file 1 [file Table_1.pdf]

Supplemental Material

Factors Associated with Utilization of Teleneurology and Other Healthcare Resources During the COVID-19 Pandemic

Benjamin R Kummer MD, Parul Agarwal PhD, Chloe Sweetnam MSc, Jessica Robinson-Papp MD PhD, Leah J Blank MD MPH, Ilana Katz Sand MD, Georges Naasan MD, Christina Palmese PhD, Joohi Jimenez-Shahed MD, Jihan Grant MD, Alison Navis MD, Laura Stein MD MPH, Shanna Patterson MD, Nathalie Jetté MD MSc

Supplemental Table 1. Diagnostic Code Groupings

ICD-10-CM Code Group	Description
C00.x-D49.x<br>D50.x-D75.x<br>D80.x-D89.x	Neoplasms
E01.x-E89.x	Endocrine, nutritional and metabolic diseases
F01.x-F05.x<br>G30.x-G32.x	Dementia and delirium
F06.x-F69.x,<br>F99.x	Mental (excluding neurodevelopmental) disorders
F79.x-F98.x	Neurodevelopmental, behavioral and emotional disorders with childhood/adolescent onset
G10.x-G14.x	Systemic atrophies of the central nervous system
G20.x-G26.x	Extrapyramidal and movement disorders
G35.x-37.x	Demyelinating nervous system disorders
G40.x<br>R56.x	Epilepsy
G43.x-G44.x<br>R51.x	Migraine and headache disorders
G45.x-46.x<br>I60.x-I69.x	Cerebrovascular disorders
G47.x<br>G80.x-83.x<br>G89.x-G99.x	Sleep and other neurological disorders
G50.x-G59.x	Neuromuscular disorders
H00.x-H57.x<br>H60.x-H93.x	Eye, ear, and adnexal disorders
I02.x-I51.x<br>I70.x-I99.x	Circulatory (excluding cerebrovascular) disorders
A00.x-B99.x<br>G00.x-G09.x	Infectious disorders
J00.x-J96.x<br>K02.x-K94.x<br>L02.x-L98.x	Respiratory, digestive, and skin disorders
M05.x-M99.x	Musculoskeletal and connective tissue disorders
R00.x-R99.x	Abnormal clinical and laboratory findings, not elsewhere classified
R25.x-R29.x	Symptoms and signs involving the nervous and musculoskeletal systems
R40.x-R46.x	Symptoms and signs involving cognition, perception, emotional state and behavior

Abbreviations: ICD-10-CM, International Classification of Diseases, 10th Edition, Clinical Modification.

Supplemental Table 2. Pre-COVID Visit Diagnoses, Stratified by TN Utilization during the COVID Period

Visit Diagnosis	Used TN (N= 4,944)	Did not use TN (N = 9,226)	p-value
Neoplasms	78 (1.6)	190 (2.1)	0.04481
Endocrine, nutritional and metabolic diseases	85 (1.7)	260 (2.8)	<0.0001
Dementia and delirium	170 (3.4)	392 (4.2)	0.01848
Mental (excluding neurodevelopmental) disorders	268 (5.4)	331 (3.6)	<0.0001
Neurodevelopmental, behavioral and emotional disorders with childhood/adolescent onset	160 (3.2)	265 (2.9)	0.22607
Systemic atrophies of the central nervous system	41 (0.8)	79 (0.9)	0.86729
Extrapyramidal and movement disorders	977 (19.8)	1,348 (14.6)	<0.0001
Demyelinating nervous system disorders	1,176 (23.8)	584 (6.3)	<0.0001
Epilepsy	579 (11.7)	1,357 (14.7)	<0.0001
Migraine and headache disorders	1,088 (22.0)	1,570 (17.0)	<0.0001
Cerebrovascular disorders	211 (4.3)	835 (9.0)	<0.0001
Sleep and other neurological disorders	180 (3.6)	515 (5.6)	<0.0001
Neuromuscular disorders	339 (6.9)	1,169 (12.7)	<0.0001
Eye, ear, and adnexal disorders	84 (1.7)	322 (3.5)	<0.0001
Circulatory (excluding cerebrovascular) disorders	48 (1.0)	231 (2.5)	<0.0001
Infectious disorders	57 (1.1)	141 (1.5)	0.06961
Respiratory, digestive, and skin disorders	123 (2.5)	399 (4.3)	<0.0001
Musculoskeletal and connective tissue disorders	503 (10.2)	1,419 (15.4)	<0.0001
Abnormal clinical and laboratory findings, NOS	455 (9.2)	1,206 (13.1)	<0.0001
Symptoms and signs involving the nervous and musculoskeletal systems	176 (3.6)	615 (6.7)	<0.0001
Symptoms and signs involving cognition, perception, emotional state and behavior	241 (4.9)	944 (10.2)	<0.0001

Data are reported as N (%), unless otherwise noted.
Abbreviations: TN, teleneurology; NOS, not otherwise specified.
"Visit diagnosis" reflects presence of one or more pre-COVID visits with this primary visit diagnosis.

Supplemental Table 3. Pre-COVID Visit Diagnoses, Stratified by Loss to Follow-Up during the COVID Period

Visit Diagnosis	Lost to follow-up (N = 4,997)	Not lost to follow-up (N= 9,173)	p-value
Neoplasms	91 (1.8)	177 (1.9)	0.6506
Endocrine, nutritional and metabolic diseases	129 (2.6)	216 (2.3)	0.4026
Dementia and delirium	194 (3.9)	368 (4.0)	0.7060
Mental (excluding neurodevelopmental) disorders	185 (3.7)	414 (4.5)	0.0219
Neurodevelopmental, behavioral and emotional disorders with childhood/adolescent onset	204 (4.1)	221 (2.4)	<0.0001
Systemic atrophies of the central nervous system	46 (0.9)	74 (0.8)	0.4798
Extrapyramidal and movement disorders	506 (10.1)	1,819 (19.8)	<0.0001
Demyelinating nervous system disorders	300 (6.0)	1,460 (15.9)	<0.0001
Epilepsy	723 (14.5)	1,213 (13.2)	0.0392
Migraine and headache disorders	858 (17.2)	1,800 (19.6)	0.0004
Cerebrovascular disorders	438 (8.8)	608 (6.6)	<0.0001
Sleep and other neurological disorders	280 (5.6)	415 (4.5)	0.0045
Neuromuscular disorders	661 (13.2)	847 (9.2)	<0.0001
Eye, ear, and adnexal disorders	195 (3.9)	211 (2.3)	<0.0001
Circulatory (excluding cerebrovascular) disorders	102 (2.0)	177 (1.9)	0.6476
Infectious disorders	72 (1.4)	126 (1.4)	0.7445
Respiratory, digestive, and skin disorders	140 (2.8)	382 (4.2)	<0.0001
Musculoskeletal and connective tissue disorders	674 (13.5)	1,248 (13.6)	0.8458
Abnormal clinical and laboratory findings, NOS	616 (12.3)	1,045 (11.4)	0.0982
Symptoms and signs involving the nervous and musculoskeletal systems	340 (6.8)	451 (4.9)	<0.0001
Symptoms and signs involving cognition, perception, emotional state and behavior	559 (11.2)	626 (6.8)	<0.0001

Data are reported as N (%), unless otherwise noted.
Abbreviations: NOS, not elsewhere classified.
"Visit diagnosis" reflects presence of one or more pre-COVID visits with this primary visit diagnosis.

Supplemental Table 4. Patient Characteristics, Stratified by Telephone Visit Utilization during the COVID Period

Characteristic	Had a telephone visit (N = 2,093)	Did not have a telephone visit (N = 12,077)	p-value
Age, median (IQR), years	64 (48 – 75)	58 (38 – 71)	<0.001
Female sex	1,301 (62.1)	7,261 (60.1)	0.0785
Race*			<0.0001
Native American	8 (0.4)	17 (0.1)	
Asian	65 (3.1)	498 (4.1)	
Black or African American	362 (17.3)	1,882 (15.6)	
Pacific Islander	8 (0.4)	63 (0.5)	
Not reported	551 (26.3)	2,823 (23.4)	
White	1,099 (52.5)	6,794 (56.3)	
Preferred language			<0.0001
English	1,898 (90.6)	11,182 (92.6)	
Non-English	190 (9.0)	816 (6.7)	
Unknown	5 (0.2)	79 (0.6)	
Insurance coverage			<0.0001
Commercial	555 (26.5)	5,351 (44.3)	
Medicaid	362 (17.3)	1,493 (12.4)	
Medicare	1,033 (49.3)	4,400 (36.4)	
Other	44 (2.1)	312 (2.6)	
Unknown	99 (4.7)	521 (4.3)	
ZIP code AGI per household, median, $US	79,292 (53,526 – 173,596)	88,431 (54,845 – 180,347)	0.049
Charlson-Deyo comorbidity index			<0.0001
0	1,232 (58.9)	7,889 (65.3)	
1	479 (22.9)	2,355 (19.5)	
2	188 (9.0)	903 (7.5)	
≥ 3	194 (9.3)	930 (7.7)	
Health utilization during COVID19 period**			
≥ 1 TN visit	707 (33.8)	4,237 (35.1)	0.258
≥ 1 office visit	918 (43.9)	3,659 (30.3)	<0.001
≥ 1 ED visit	298 (14.2)	1,016 (8.4)	<0.001
≥ 1 hospital admission	210 (10.0)	675 (5.6)	<0.001
Lost to follow-up	0 (0.0)	4,997 (35.3)	<0.001

Data are reported as N (%), unless otherwise noted.

*Race variable is imputed as described in Methods. **Percentages may not add to 100% since categories are not mutually exclusive.

Abbreviations: COVID19, coronavirus disease 2019; IQR, interquartile range; AGI, annual gross income.

Supplemental Table 5. Patient Characteristics, Stratified by Office Visit Utilization during the COVID Period			
**Characteristic**	**Had an office visit (N = 4,577)**	**Did not have an office visit (N = 9,593)**	**p-value**
Age, median (IQR), years	62.0 (48.0 – 74.0)	56.0 (36.0 – 71.0)	<0.001
Female sex	2,827 (61.8)	5,735 (59.8)	0.0240
Race*			<0.0001
Native American	9 (0.2)	16 (0.2)	
Asian	177 (3.9)	386 (4.0)	
Black or African American	718 (15.7)	1,526 (15.9)	
Pacific Islander	29 (0.6)	41 (0.4)	
Not reported	1,102 (24.1)	2,271 (23.7)	
White	2,541 (55.5)	5,352 (55.8)	
Preferred language			0.0028
English	4,180 (91.3)	8,900 (92.8)	
Non-English	373 (8.1)	633 (6.6)	
Unknown	24 (0.5)	60 (0.6)	
Insurance coverage			<0.0001
Commercial	1,547 (33.8)	4,359 (45.4)	
Medicaid	640 (14.0)	1,215 (12.7)	
Medicare	2,107 (46.0)	3,326 (34.7)	
Other	104 (2.3)	252 (2.6)	
Unknown	179 (3.9)	441 (4.6)	
ZIP code AGI per household, median, $US	87,535 (53,526 – 179,965)	86,910 (55,587 – 180,347)	0.693
Charlson-Deyo comorbidity index			<0.0001
0	2,792 (61.0)	6,329 (66.0)	
1	1,000 (21.8)	1,834 (19.1)	
2	392 (8.6)	699 (7.3)	
≥ 3	393 (8.6)	731 (7.6)	
Health utilization during COVID19 period**			
≥ 1 TN visit	1,645 (35.9)	3,299 (34.4)	0.073
≥ 1 telephone visit	918 (20.1)	1,175 (12.2)	<0.001
≥ 1 ED visit	555 (12.1)	759 (7.9)	<0.001
≥ 1 hospital admission	366 (8.0)	519 (5.4)	<0.001
Lost to follow-up	0 (0.0)	4,977 (52.1)	<0.001

Data are reported as N (%), unless otherwise noted.

*Race variable is imputed as described in Methods. **Percentages may not add to 100% since categories are not mutually exclusive.

Abbreviations: COVID19, coronavirus disease 2019; IQR, interquartile range; AGI, annual gross income.

Supplemental Table 6. Patient Characteristics Stratified by Utilization of ED Visits during the COVID Period

Characteristic	Presented to ED (N = 1,314)	Did not present to ED (N = 12,856)	p-value
Age, median (IQR), years	64.0 (48.0 – 75.0)	58.0 (38.0 – 72.0)	<0.001
Female sex	832 (63.3)	7,730 (60.1)	0.0243
Race*			<0.0001
Native American	2 (0.2)	23 (0.2)	
Asian	45 (3.4)	519 (4.0)	
Black or African American	363 (27.6)	1,881 (14.6)	
Pacific Islander	5 (0.4)	65 (0.5)	
Not reported	457 (34.7)	2,917 (22.7)	
White	443 (33.7)	7,451 (58.0)	
Preferred language			<0.0001
English	1,133 (86.2)	11,947 (92.9)	
Non-English	179 (13.6)	827 (6.4)	
Unknown	2 (0.1)	82 (0.6)	
Insurance coverage			<0.0001
Commercial	235 (17.9)	5,671 (44.1)	
Medicaid	300 (22.8)	1,555 (12.1)	
Medicare	670 (51.0)	4,763 (37.0)	
Other	32 (2.4)	324 (2.5)	
Unknown	77 (5.9)	543 (4.2)	
ZIP code AGI per household, median, $US	68,403 (51,246 - 173,596)	88,813 (55,881 –180,347)	<0.001
Charlson-Deyo comorbidity index			<0.0001
0	510 (38.8)	8,611 (67.0)	
1	400 (30.4)	2,434 (18.9)	
2	200 (15.2)	891 (6.9)	
≥ 3	204 (15.5)	920 (7.1)	
Health utilization during COVID19 period**			
≥ 1 TN visit	351 (26.7)	4,593 (35.7)	<0.001
≥ 1 telephone visit	298 (22.7)	1,795 (14.0)	<0.001
≥ 1 office visit	555 (42.2)	4,022 (31.3)	<0.001
≥ 1 hospital admission	623 (47.4)	262 (2.0)	<0.001
Lost to follow-up	0 (0.0)	4,997 (38.9)	<0.001

Data are reported as N (%), unless otherwise noted.

*Race variable is imputed as described in Methods. **Percentages may not add to 100% since categories are not mutually exclusive.

Abbreviations: ED, emergency department; COVID19, coronavirus disease 2019; IQR, interquartile range; AGI, annual gross income.

Supplemental Table 7. Patient Characteristics, Stratified by Utilization of Hospital Admissions during the COVID Period			
**Characteristic**	**Hospitalized (N = 885)**	**Not hospitalized (N = 13,285)**	**p-value**
Age, median (IQR), years	66.0 (50.0 – 77.0)	58.0 (39.0 – 72.0)	<0.001
Female sex	503 (56.8)	8,059 (60.7)	0.027
Race*			<0.0001
Native American	2 (0.2)	23 (0.2)	
Asian	32 (3.6)	531 (4.0)	
Black or African American	214 (24.1)	2031 (15.3)	
Pacific Islander	4 (0.5)	66 (0.5)	
Not reported	265 (30.0)	3,108 (23.4)	
White	368 (41.6)	7,526 (56.7)	
Preferred language			
English	765 (86.4)	12315 (92.7)	<0.001
Non-English	119 (13.4)	887 (6.7)	
Unknown	1 (0.1)	83 (0.6)	
Insurance coverage			
Commercial	181 (20.5)	5725 (43.1)	<0.001
Medicaid	152 (17.2)	1703 (12.8)	
Medicare	489 (55.3)	4944 (37.2)	
Other	9 (1.0)	347 (2.6)	
Unknown	54 (6.1)	566 (4.3)	
ZIP code AGI per household, median, $US	71,181 (53,526 – 173,596)	88,124 (54,665 – 180,347)	0.003
Charlson-Deyo comorbidity index			
0	317 (35.8)	8,804 (66.3)	<0.001
1	257 (29.0)	2,577 (19.4)	
2	134 (15.1)	957 (7.2)	
≥ 3	177 (20.0)	947 (7.1)	
Health utilization during COVID19 period **			
≥ 1 TN visit	304 (34.4)	4,640 (34.9)	0.755
≥ 1 telephone visit	210 (23.7)	1,883 (14.2)	<0.001
≥ 1 office visit	366 (41.4)	4,211 (31.7)	<0.001
≥ ED visit	623 (70.4)	691 (5.2)	<0.001
Lost to follow-up	0 (0.0)	4,997 (37.6)	<0.001

Data are reported as N (%), unless otherwise noted.
*Race variable is imputed as described in Methods. **Percentages may not add to 100% since categories are not mutually exclusive.
Abbreviations: COVID19, coronavirus disease 2019; IQR, interquartile range; AGI, annual gross income.
